# Fully Endoscopic Microvascular Decompression: Our Early Experience

**DOI:** 10.1155/2013/739432

**Published:** 2013-09-03

**Authors:** Casey H. Halpern, Shih-Shan Lang, John Y. K. Lee

**Affiliations:** Department of Neurosurgery, Hospital of the University of Pennsylvania, 235 South Eighth Street, Philadelphia, PA 19106, USA

## Abstract

*Background*. Microvascular decompression (MVD) is a widely accepted treatment for neurovascular disorders associated with facial pain and spasm. The endoscope has rapidly become a standard tool in neurosurgical procedures; however, its adoption in lateral approaches to the posterior fossa has been slower. The endoscope is used primarily to assist conventional microscopic techniques. We are interested in developing fully endoscopic approaches to the cerebellopontine angle, and here, we describe our preliminary experience with this procedure for MVD. *Methods*. A retrospective review of our two-year experience from 2011 to 2012, transitioning from using conventional microscopic techniques to endoscope-assisted microsurgery to fully endoscopic MVD, is provided. We also reviewed our preliminary outcomes during this transition. *Results*. There was no difference in the surgical duration of these three procedures. In addition, the majority of procedures performed in 2012 were fully endoscopic, suggesting the ease of incorporating this solo tool into practice. Pain outcomes of fully endoscopic MVD appear to be very similar to those of both conventional and endoscope-assisted MVDs. Complications occurred in all groups at equally low rates. *Conclusion*. Fully endoscopic MVD is both safe and effective. By enhancing visualization of structures within the cerebellopontine angle, endoscopy may prove to be a valuable adjunct or alternative to conventional microscopic approaches.

## 1. Introduction

Microvascular decompression (MVD) is a highly effective surgical treatment of neurovascular disorders associated with facial pain and spasm (e.g., trigeminal neuralgia, hemifacial spasm) [1, 2]. However, failure to relieve symptoms associated with these conditions can occur due to poor visualization of the offending vascular contact at the root entry zone or medial vascular compression [3]. The endoscope has quickly become a standard operative tool in minimally invasive neurosurgery of the sella and ventricular system due to the panoramic views and bright illumination [4–6]. Similarly, advantages of the endoscope in visualizing structures within the cerebellopontine angle (CPA), including nerve cleavage planes and vascular anatomic features, have been noted in addition to smaller exposures and less cerebellar or brainstem retraction than conventional microscopy [7, 8]. The endoscope has been reported to be used as a standard adjunct to conventional MVD in a number of institutions, but rarely as a solo technique [9, 10]. This paper outlines our approach to fully endoscopic MVD (E-MVD) for the treatment of multiple neurovascular compression syndromes. We report our preliminary experience with this technique since incorporating the endoscope into our practice in early 2011.

## 2. Surgical Technique

### 2.1. Patient Positioning and Equipment Setup

The surgical technique has been previously described by us and others [11, 12]. Briefly, all operations are performed under general anesthesia. Prior to positioning, the patient's head is secured in a Mayfield clamp. The body is then turned in a full lateral position with an axillary role. The patient is taped down securely, and the head is flexed approximately two fingerbreadths from the sternum and rotated 70–80 degrees away from the side of the operation in order to maintain the vertex parallel to the floor. In this position, the cranial nerve 7-8 bundle is more inferior to the trigeminal nerve.

**Figure 1:**
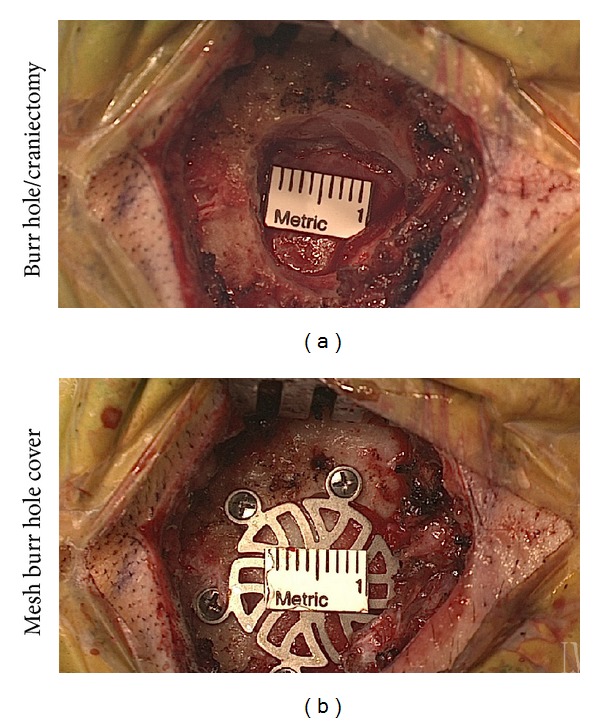
Endoscopic view of burr hole/craniectomy site (a) with subsequent closure and placement of titanium mesh burr hole cover (b).

A small region of hair is shaved postauricularly, and a 4–6 cm linear incision is made just inferior to the junction of the transverse and sigmoid sinuses and approximately one centimeter behind the patient's hairline. The burr hole for the craniectomy is placed just posterior to the most superior aspect of the insertion of the digastric muscle. Once the inferior border of the transverse sinus and posterior border of the sigmoid sinus are identified following minimal craniectomy (Figures 1(a) and 1(b)), a 1 cm C-shaped dural opening is made. The dural flap is retracted anteriorly and reflected against the sinuses. None of these techniques differ from that of the conventional microscopic approach.

**Figure 2:**
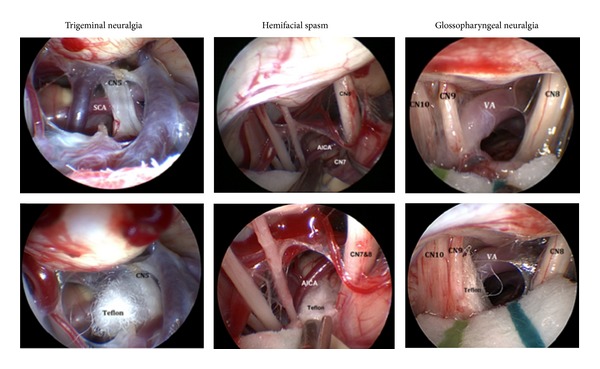
Endoscopic view of the vascular compression associated with cases of trigeminal neuralgia, hemifacial spasm, and glossopharyngeal neuralgia. The left panel demonstrates compression of CN 5 by the superior cerebellar artery (top) with subsequent decompression with Teflon (bottom). The middle panel illustrates compression of CN 7 by the anterior inferior cerebellar artery (top). Note that CN 7 is found on the inferior aspect of CN 8, which requires decompression deep to the CN 7-8 complex (bottom). The right panel demonstrates a case of glossopharyngeal neuralgia caused by vertebral artery contact (top) and the associated decompression (bottom). Cranial nerve (CN); superior cerebellar artery (SCA); anterior inferior cerebellar artery (AICA); vertebral artery (VA).

At this point, however, the endoscope that is attached to the Mitaka Pneumatic Arm (Mitaka Kohki Co.) is brought in. Our preference is to use the 2.7 mm zero-degree endoscope upfront (Storz; Culver City, CA, USA). This smaller diameter endoscope maximizes the amount of working space that is needed for the other instruments, thus minimizing instrument conflict and brain retraction (Figure 2). In conjunction with a high-definition camera, this system provides excellent visualization. Angled endoscopes are also utilized to facilitate identification of vascular contacts at the root entry zone and to visualize the trigeminal nerve medial to a low and prominent petrous ridge.

### 2.2. Endoscopic Microvascular Decompression

Next, the endoscope is inserted through the dural opening with minimal retraction on the cerebellum. The arachnoid sheath around the cranial nerve 9–11 bundle is dissected with sharp scissors, and the cerebrospinal fluid is drained, enhancing visualization of structures within the CPA. Using a combination of bipolar coagulation of minor vessels and blunt dissection, the arachnoid around the trigeminal nerve is then lysed, and the vascular anatomy is inspected. The offending vessel is then mobilized, and decompression is achieved with a Teflon pad placed between the offending vessel and trigeminal nerve. The 30-degree endoscope has been found to be the most useful in identifying occult vascular structures at the root entry zone (Figure 2), venous compression, as well as the nervus intermedius in the case of geniculate neuralgia. 

## 3. Patient Population

From September 2010 to November 2012, 70 patients (M/F: 24/46) with the diagnosis of medically refractory trigeminal neuralgia (TGN), hemifacial spasm (HFS), glossopharyngeal neuralgia (GPN), or geniculate neuralgia (GN) were seen in the neurosurgery clinic for preoperative evaluation. Prior to this period, all patients who underwent microvascular decompression by the senior author (John Y. K. Lee) had undergone a purely microscopic surgical procedure. In the first half of this experience (9/2010 to 12/2011), 14 MVDs, 8 endoscope-assisted (EA)-MVDs, and 16 purely E-MVDs were performed. In the next half of this experience (January 2012 to November 2012,) 9 MVDs, 1 EA-MVD, and 22 purely E-MVDs were performed. Hence, the great majority of procedures performed were purely endoscopic. The decision to perform a microscopic procedure versus an endoscope-assisted procedure and purely endoscopic procedure reflected the comfort of the surgeon as well as the availability of equipment. 

The great majority of procedures were performed for TGN (*n* = 62), whereas HFS (*n* = 5), geniculate neuralgia (*n* = 2), and GPN (*n* = 1) were less well represented in this cohort (see Table 1). One patient in the conventional MVD group had been treated previously with cyber knife surgery and MVD. Three patients in the EA-MVD group were previously treated with gamma knife surgery, and one surgical exploration without decompression. Three patients in the E-MVD had previous gamma knife surgery treatments, and five had prior conventional MVD. 

EA-MVD procedures were performed as part of a gradual transition to a fully endoscopic procedure. Nearly 60 percent of the E-MVD, and only 1 of the 9 EA-MVD cases took place in 2012, reflecting the rapidity that this solo technique was adopted at our institution. Of interest, there was no apparent difference in surgical durations between these three treatment groups. All surgeries were performed by the senior author (John Y. K. Lee).

## 4. Surgical Findings

An offending vessel was identified in all cases except in one of the fully endoscopically treated patients, for which Teflon was placed between the arachnoid and nerve, and the case of geniculate neuralgia in which sectioning of the nervus intermedius was performed. Arterial compression was identified in 14 of the 23 MVD patients, 5 of the 9 EA-MVD patients, and 31 of the 38 E-MVD patients. Venous compression was identified in 12 of the MVD patients, 3 of the 9 EA-MVD patients, and 12 of the E-MVD patients. Of the 62 patients with TGN, only 2 did not have any vessel identified. The rate of vascular contact was similar between the 3 groups of MVD, EA-MVD, and E-MVD. Only one patient in each group EA-MVD and E-MVD was not shown to have a vessel contact; see Figure 2.

A surgical neurolysis was performed in a minority of patients (total *n* = 12) and was undertaken either with a round knife along the fascicles of the nerve (*n* = 10) or with direct injection of 0.2 cc of glycerol (*n* = 2). This was performed at the discretion of the senior surgeon (John Y. K. Lee) based on intraoperative findings, such as insignificant vascular compression. The rate of neurolysis was 26% in the MVD group, 11% in the EA-MVD group, and 16% in the E-MVD group. Neurolysis was not performed in cases of HFS. 

## 5. Outcomes/Followup

Patients were followed up for approximately 2–3.5 months following surgery (see Table 1). We classified pain according to the Barrow Neurological Institute scale for pain in trigeminal neuralgia, and we considered success if BNI score was between 1 (no pain, no meds), 2 (occasional pain, no meds), and 3 (some pain, adequately controlled). Using these criteria for the 62 patients with trigeminal neuralgia, we identified only 5 patients of 62 who had BNI class IV (some pain, not adequately controlled) or class V (severe pain or no relief). One of these patients was in the microscope only arm (1/20 = 5%). Four of these patients were in the endoscope only arm (4/31 = 11%), and none of the patients in the EA-MVD group were BNI class IV or V (0/7 = 0%). This was not a statistically different difference between the three groups (MVD, EA-MVD, and E-MVD), using the Kruskal Wallis test (*P* = 0.5018); see Table 1.

Of the five patients with HFS, all five had an excellent outcome with complete resolution of their hemifacial spasm. The two patients with geniculate neuralgia did not experience significant benefit and were classified as BNI class IV at last followup. The patient with glossopharyngeal neuralgia had complete relief of pain.

The conventionally treated patient who had previous cyber knife surgery did not respond to MVD. Three of the 8 E-MVD patients who had previous gamma knife surgery or MVD did report 100 percent resolution of pain. Three of the 4 EA-MVD patients who were previously treated procedurally also reported complete resolution of their symptoms.

Complications were unusual overall. In the TGN cohort, there was one wound infection (MVD), one temporary facial palsy (MVD), which required temporary gold weight but returned to normal by 9 months, and one CSF otorrhea, which required lumbar drainage for five days but sealed on its own (E-MVD). The total complication rate was 3/62 = 4.8% complication rate. None of the complications appear to have been directly related to endoscopic technique. In the hemifacial spasm cohort, there was one temporary facial palsy, which improved at six months to HB grade I (EA-MVD). This was in a patient with very severe hemifacial spasm and significant tonus, who had received prior Botox therapy. In the three patients with glossopharyngeal and geniculate neuralgia, no complications were identified.

Patients who required reoperation generally did well. The one patient in the conventional MVD group who was previously explored (no Teflon was found intraoperatively) had complete resolution of symptoms (BNI 1) following MVD. One patient in the EA-MVD who had a previous MVD without success also saw complete resolution of his symptoms. Three of the 6 patients with previous MVD who underwent E-MVD had a BNI score of 1 at followup. These data are supported by previous findings in which reoperation is both safe and frequently effective for either persistent or recurrent facial pain [3]. 

We performed a simple stepwise forward logistic regression analysis using the primary outcome measure of a BNI class score of three or better. We included the following variables: gender, presence of vein, artery, use of endoscope, and neurolysis. Surprisingly, the strongest predictor of success was the performance of a neurolytic procedure, although the overall *P* value of the model only approached statistical significance at *P* = 0.0593 (STATA 10). This finding forced us to look at the results in patients undergoing neurolysis. Although the act of neurolysis was a significant predictor in our stepwise model, it was not a predictor at face value. Two of 12 patients who underwent neurolysis had a poor outcome versus 3 of 50 patients who did not undergo a neurolysis. (*P* = 0.2245 Fisher's exact).

## 6. Discussion

Our preliminary experience with fully endoscopic MVD supports the safety, feasibility, and potential benefits of this approach for a wide variety of neurovascular syndromes. Although we did not demonstrate superiority of the fully endoscopic approach, we did not find any statistically significant difference when compared to the microscopic procedure. It is important to note that no difference was found despite the relatively short period of time following introduction of the endoscope into our routine practice. There was also no difference in operative time. Hence, we believe that the use of the endoscope is safe for microvascular decompression, and at least as effective as a microscopic procedure. 

The use of the endoscope in the posterior fossa, however, offers additional benefits to skull base surgeons. Once the surgeon becomes accustomed to the technique of operating with the endoscope using the view from the monitor, it opens up the possibility of operating with angled scopes, which may allow safe access to structures not previously seen using the standard operative microscope. We believe that extended retrosigmoid approaches to more difficult areas of the brain will become more likely. For example, an extended retrosigmoid approach via a suprameatal approach to trigeminal schwannomas can be facilitated [13]. In addition, endoscopic supracerebellar transtentorial approaches to the medial posterior temporal lobe are a possibility [14]. This will allow the skull base surgeon improved access to regions of the brain that have traditionally required either more bone removal or intraparenchymal corridors. Facility with the simple microvascular decompression will allow the surgeon to tackle more complex pathology [15], perhaps even minimizing the morbidity associated with petroclival meningiomas in the future.

## 7. Conclusion

Endoscopic microvascular decompression is a safe, feasible and effective procedure for cure of TGN, hemifacial spasms and other cranial nerve disorders. Our two-year experience summarizes the transition from conventional microscopic surgery to a fully endoscopic procedure, demonstrating the ease and safety of incorporating this tool into practice as a solo instrument. Expanding this experience from neurovascular syndromes to cerebellopontine angle tumors represents the next step in the expanding era of minimally invasive endoscopic neurosurgery.

## Figures and Tables

**Table 1:** Patient summary.

	Diagnosis (*N*)	Mean surgical duration (±SD)	Mean followup time	Vascular contact (*N*)	BNI class I to III
MVD	TGN (20) HFS (2) GPN (1),	129 ± 36	62	Artery (11) Vein (12)	19/20 (95%)

EA-MVD	TGN (7) HFS (2)	137 ± 29	77	Artery (3) Vein (6)	7/7 (100%)

E-MVD	TGN (35) HFS (1) GN (2)	130 ± 32	107	Artery (26) Vein (12)	31/35 (89%)

Microvascular decompression (MVD); endoscope-assisted microsurgery (EA-MVD); fully endoscopic microvascular decompression (E-MVD); Trigeminal neuralgia (TGN); hemifacial spasm (HFS); glossopharyngeal neuralgia (GPN); geniculate neuralgia (GN).
